# Characterization of Esterase Genes Involving Malathion Detoxification and Establishment of an RNA Interference Method in *Liposcelis bostrychophila*

**DOI:** 10.3389/fphys.2020.00274

**Published:** 2020-03-27

**Authors:** Dan-Dan Wei, Wang He, Zhe-Qing Miao, Yan-Qing Tu, Lei Wang, Wei Dou, Jin-Jun Wang

**Affiliations:** ^1^Key Laboratory of Entomology and Pest Control Engineering, College of Plant Protection, Southwest University, Chongqing, China; ^2^Academy of Agricultural Sciences, Southwest University, Chongqing, China

**Keywords:** booklice, stored product pests, microsatellite, detoxification enzyme, RNAi

## Abstract

Esterases (ESTs) play important roles in metabolizing various physiologically endogenous and exogenous compounds, and various environmental xenobiotics in insects. The psocid, *Liposcelis bostrychophila* is a major pest of stored products worldwide and rapidly develops resistance to commonly insecticides. However, the involvement of ESTs in insecticide metabolization and the application of RNAi approach in psocids have not been well elucidated. In this study, we characterized four *LbEST* genes and investigated the transcriptional levels of these genes at different developmental stages and under different insecticides exposures to assess their potential roles in response to insecticides. The four *LbESTs* contain a catalytic triad (Ser-His-Glu) linked to an oxyanion hole and acyl pocket involved in substrate stabilization during its hydrolysis. Synergism observed with the esterase-inhibitor DEF suggests the involvement of esterases in malathion detoxification. *LbESTs* were expressed during the whole of developmental stages, but predominant abundance in the first nymphal instar and adult stage. The mRNA level of three *LbEST* genes (except for *LbEST4*) was induced (1.29- to 5.60 fold) in response to malathion or deltamethrin exposures, indicating that these esterases are involved in the detoxification process. Silencing of *LbEST1*, *LbEST2* or *LbEST3* through dsRNA feeding led to a higher mortality of psocids upon the malathion treatment compared to controls (1.83 to 2.69-fold), demonstrating that these esterase genes play roles in malathion detoxification in *L. bostrychophila*. Our study provides new evidence for understanding of the function and regulation mechanism of esterases in *L. bostrychophila* in insecticide detoxification. The current study also suggests that the present RNAi method could be applied for gene functional studies in psocids.

## Introduction

The psocids from *Liposcelis* genus (Psocoptera: Liposcelididae), are stored-product pests worldwide that pose new risks for global food safety and security (Nayak et al., 2014). Recently, *Liposcelis bostrychophila* (Badonnel) has gained important recognition due to their parthenogenic reproduction, rapid adaptation, and increased worldwide distribution. Further, as stored-product pests, psocids are not easily managed (Wei et al., 2011). Currently, the primary means control depends on chemical insecticides. Intensive and unreasonable use of insecticides has caused high levels of resistance in psocids, which has created a serious obstacle for the chemical control of these pests (Ding et al., 2004; Nayak et al., 2014).

Many early and unsuccessful attempts to control psocids may be due to the apparent resistance to insecticides. Treatments failed to suppress populations of *L. bostrychophila* and *L. entomophila* when permethrin, fenitrothion and malathion were mixed with rice (Nayak et al., 2014). Variable degrees of tolerance of *L. bostrychophila* to pyrethroids (permethrin, cypermethrin and deltamethrin), organophosphates (fenitrothion, malathion, and pirimiphos-methyl) and insect growth regulators (methoprene and fenoxycarb) were detected (Leong and Ho, 1994; Nayak, 2010). Furthermore, bacterium-derived spinosad, imidacloprid, and diatomaceous earth are also not suitable against any *Liposcelis* species (Nayak and Daglish, 2006; Athanassiou et al., 2009, 2010). In fact, different *Liposcelis* species usually exhibit various degrees of tolerance to chemical treatments, and this has made it difficult to control in the field, since psocid infestations often involve more than one species. The above studies also demonstrate that *L. bostrychophia* possess naturally higher tolerance to contact insecticides compared with other insect pests of stored products. However, the molecular mechanisms underlying tolerance or resistance to insecticides in *L. bostrychophia* have not been well characterized.

Like other insects, multiple resistance mechanisms in *Liposcelis* psocids have been reported (Wei et al., 2014). Metabolic resistance (e.g., P450s, GSTs) (Jiang et al., 2012; Jing et al., 2017) and target-site insensitivity (e.g., nicotinic acetylcholine receptor, acetylcholinesterase) (Tang et al., 2009; Wu et al., 2010) are the two main mechanisms involved in the development of insecticide resistance in psocids. In *L. bostrychohila*, the detoxifying enzymes in insecticide resistance have been widely studied from synergist bioassays to molecular level. For example, previous study suggested that CYP4CB1 and CYP4CC1 are possibly associated with deltamethrin and paraoxon-methyl metabolism in *L. bostrychohila* based on induction profiles, and CYP4CD1 is potentially involved in aldicarb metabolism (Jiang et al., 2012). Transcriptome studies showed that P450s, GSTs, and ESTs may play a role in insecticide tolerance after short-term exposure to deltamethrin and malathion (Dou et al., 2013). Also enhanced carboxylesterase activities may contribute to insecticide tolerance or resistance in booklice (Wang et al., 2004). Elevated GST activities were also confirmed in DDVP and PH_3_-resistant strains of *L. bostrychohila* by enzymatic assays (Dou et al., 2009). In addition, insecticide synergists are a useful tool to determine whether the metabolic resistance or increased insecticides toxicity is present in insects (Gonzalez-Morales and Romero, 2019). For example, effectiveness of synergized-pyrethrins with piperonyl butoxide (PBO) was enhanced against several *Liposcelis* species (Nayak, 2010).

ESTs are ubiquitous in all organisms as a major class of detoxification genes and are involved in many metabolic reactions, neurogenesis and developmental regulations (Panini et al., 2016). As a large group of phase 1 metabolic enzymes, they play crucial roles in insecticide detoxification through either sequestration or direct metabolism of a number of insecticides, including pyrethroids, organophosphates and carbamates. ESTs based resistance mechanisms can be mediated by quantitative or qualitative changes, leading to the overexpression of these enzymes or modifications of their structures (Cui et al., 2015). Esterase overexpression can be due to either gene amplification or up-regulation, or a combination of both. Based on their physiological and biochemical roles, ESTs can be divided into three classes: the first class (clades A-C) usually contains primarily intracellular esterases with dietary/detoxification functions, and which are involved in resistance to many insecticides; the second class (clades D-H) contains catalytically active and secreted esterases that involved in pheromone and juvenile hormone (JH) degradation; the third class (clades I-M) contains esterases with neuro/developmental function, including acetylcholinesterase (Oakeshott et al., 2005).

Post-transcriptional gene silencing by RNA interference (RNAi) is an effective approach that has been applied to explore gene function and pest control in arthropod (Gu and Knipple, 2013; Niu et al., 2018). For example, RNA pesticide (dsATPD + dsCHS1) shows a great potential for the soybean aphid management (Yan et al., 2019). Moreover, RNAi can aid in uncovering the roles of genes in insecticide detoxification and resistance (Kim et al., 2015). Silencing an esterase gene in *Aphis gossypii* decreased the activity of esterase, and increased the sensitivity of aphids to insecticides (Gong et al., 2014). However, to our knowledge, RNAi has not been used to verify gene function in psocids. Furthermore, there is limited information about esterase activity and their expression profiles in response to insecticide exposure in psocids. Hence, this study aimed to clarify the relative contribution of esterase genes associated with insecticide metabolism in stored product psocids through transcript level analysis and use of RNAi.

## Materials and Methods

### Insects

*Liposcelis bostrychophila* was sampled at grain storage facilities in Beibei, Chongqing, China in 2002, and was fed on an artificial diet consisting of whole-wheat flour, skimmed milk, and yeast powder (10:1:1) in an incubator at 27 ± 0.5°C, 75–80% RH and a scotoperiod of 0:24 (L: D) h. In laboratory culture conditions, 100 plastic vials (1 cm high by 2.4 cm diameter) with diet were used for collecting uniform eggs. Fifty adults were placed into each vial, and 48 h later, the adults were removed. Developmental periods of 5, 11, 15, 19, 22, and 26 days were used for collecting the egg, 1^st^, 2^nd^, 3^rd^, 4^th^ stadium nymphs and adults at the culture conditions, respectively.

### Insecticides Bioassays and Synergistic Experiment

Insecticide bioassays were performed as previously published work with slight modifications (Cheng et al., 2004). Two insecticides, including malathion (purity 89.5%) (Organophosphorus, ChemService, West Chester, PA) and deltamethrin (purity 99%) (Pyrethroids, Sigma Aldrich, St. Louis, MO) were dissolved with acetone as stock solutions, and then diluted them to a series of concentrations. For each insecticide solution, after making an layer of insecticide (300 μL) in a glass Petri dish (6-cm-diameter), thirty test adults were exposed for 30 min, and then transferred to a fresh glass dish. The mortality was evaluated after 24 h. As controls, psocids were treated with acetone alone. All tests were performed at 27 ± 0.5°C (75–80% RH, dark condition) and replicated three times. For the synergism bioassays, synergist of 97% TPP (Institute for Control of Agrochemicals, Sichuan Province, China), 97% DEM (Institute for Control of Agrochemicals, Sichuan Province, China) and 97% DEF (Sigma, Aldrich, St. Louis, MO) were dissolved in acetone, and then mixed with insecticide in a final concentration of 10.6 mg/m^2^, 5.3 mg/m^2^, and 10.6 mg/m^2^, respectively. The above synergism bioassays were conducted using the same method as the insecticide bioassay.

### Insecticide Exposures

The 3 to 5 days old adults were treated by two insecticides (malathion and deltamethrin) based on calculated LC_50_s. Adults exposed to acetone were used as controls. After 30 min exposure, the insects were transferred into a new glass vial with a small amount of diet under our normal rearing conditions, and then after 24, 48, and 72 h exposure, alive adults were collected from each replicate and frozen using liquid nitrogen for RNA extraction. All tests were performed at 27 ± 0.5°C (75–80% RH, dark condition) and replicated three times.

### Enzymatic Activity Determination

One thousand adults were exposed to insecticides in Petri dishes at LC_50_ values for 30 min. Adults treated by acetone were used as controls. After 30 min, all insects were transferred into new Petri dishes under normal conditions. After 6, 12, 24, 36, and 48 h of the time point, surviving adults were homogenized on ice in 1 mL ice-cold PBS buffer (0.04 mol/L, pH 7.0). The crude homogenates were centrifuged at 10,000 × *g* for 15 min at 4°C. The supernatants were used as the enzyme sources for assaying esterase activity. Bradford’s method was used to measure the protein concentration (Bradford, 1976) utilizing bovine serum albumin as a standard.

Esterase activity was measured using α-naphthyl acetate (α-NA) and β-naphthyl acetate (β-NA) as a model substrate by Van Asperen’s method with minor modifications (Asperen, 1962). A 175 μL reaction system that contained 125 μL α-NA/β-NA (0.6 × 10^–4^ mol/L) and 50 μL crude enzyme (0.04 M, pH 7.0 PBS as control) was incubated at 30°C for 10 min, and then 25 μL color developing agent (mixed as follows: 5% SDS: 1% fast blue B salt = 5:2 v/v) was added to stop the reaction. OD values were recorded at 600 nm (β-NA at 555 nm) using a microplate reader (Bio-Rad, Hercules, CA) after 10 min of incubation at 30°C in the dark. A standard curve was plotted using the concentration of α-naphthol as the abscissa and OD values as the ordinate. The amount (M) of decomposed α-NA by esterase was calculated based on standard curve. The activity of esterase was expressed in nmol of α-naphthol mg of protein^–1^ min^–1^.

### RNA Extraction and cDNA Synthesis

Total RNA was extracted from insecticide-exposure adults, different developmental stages, as well as dsRNA treated adults of *L. bostrychophila* with TRIzol Reagent (Invitrogen Life Technologies, Carlsbad, CA), according to the manufacturer’s instruction. The quality and concentration of the RNA were evaluated at the absorbance ratio of OD_260__/__280_ value by NanoVue UV-Vis spectrophotometer (GE Healthcare Biosciences, Uppsala, Sweden). First-strand cDNA was synthesized following the manufacturer’s instructions of PrimeScript 1st Strand cDNA Synthesis Kit (Takara Biotechnology Dalian Co., Ltd., Dalian, China). The synthesized cDNA was stored at −20°C until future use.

### PCR Confirmation, Gene Characterization and Phylogenetic Analysis

Full-length cDNAs of *LbEST1* and *LbEST2* were amplified by 5′- and 3′-RACE assays as previously reported (Tang, 2009). The *LbEST3* and *LbEST4* transcripts were obtained from transcriptome database of *L. bostrychophila* (Dou et al., 2013). The entire ORFs were amplified for the four EST genes, and primers were shown in Table 1. PCR was carried out using the following conditions: 3 min denaturation at 95°C, 34 cycles of 95°C for 30 s, 50–60°C (according to the melting temperature of primer) for 40 s, 72°C for 1 min, and a final extension at 72°C for 10 min. The total volume of PCR was 25 μL with 2.5 μL 10 × PCR buffer (Mg^2+^ free), 2.0 μL dNTPs (25 mM), 2.5 μL Mg^2+^ (25 mM), 1 μL cDNA templates (300–500 ng/μL), 1 μL each of primers (0.1 mM) and 15 μL ddH_2_O. All the reactions were catalyzed by rTaq^TM^ polymerase (Takara, Dalian, China), and were performed in a C1000^TM^ thermal cycler (BIO-RAD, Hercules, CA). The amplified products were gel purified with the Gel Extraction Mini Kit (Watson Biotechnologies, Shanghai, China). Then, purified products were cloned into a pGEM-T easy vector (Promega, Fitchburg, MA), and introduced into *Escherichia coli* (Trans5α, Beijing TransGen Biotech, Beijing, China) following by ampicillin selection. The plasmid clones was sequenced at BGI, Beijing, China.

**TABLE 1:** Primers used for cloning, qPCR, and double-stranded RNA (dsRNA) synthesis of four esterase genes in *Liposcelis bostrychophila.*

**Application of primers**	**Primer names and sequences (5′to 3′)**	**Product size (bp)**
ORF confirmation	*LbEST1*-F: GCGGGGAGATTGATAGTGT	1827
	*LbEST1*-R: TGGATATACAGTAGAGGAACGT	
	*LbEST2*-F: GCGGGACTCGATAAGTGTC	2411
	*LbEST2*-R: TTCTCGTGGGGTGGGATTT	
	*LbEST3*-F: GATGAAGTGTGCAGTAATTCTT	1782
	*LbEST3*-R: TGCCCTCTTCTTCATTAGAG	
	*LbEST4*-F: ACAAACGACAAATGGCTCTTCA	1824
	*LbEST4*-R: GTACCCGTAATCAAGTTCTGCA	
qPCR	*LbEST1*-qF: CCAAAGGAACATCGCAGCAT	204
	*LbEST1*-qR: TTCCCTAACCTAAACGCCGT	
	*LbEST2*-qF: TCGGTGAAAGTGCAGGATCT	183
	*LbEST2*-qR: CCCCTTTCTTACACCCGACT	
	*LbEST3*-qF: AGCTTCGCAACAACCGGATA	196
	*LbEST3*-qR: TCGGGTTTAAAATGCCCCGA	
	*LbEST4*-qF: CCGTTCACCGTTTTTGGACC	210
	*LbEST4*-qR: TGCGATCGTTTCCCAGTTGT	
	*Lb*β*-actin*-qF: CACGGTATCGTCACCAACTG	207
	*Lb*β*-actin*-qR: AGACAATACGGCTTGGATGG	
	*Lb*α*-tubulin*-qF: AAATCGTTTCCTCGATCACG	211
	*Lb*α*-tubulin*-qR: ACCATCTGATTGGCAGGTTC	
dsRNA synthesis	*LbEST1*-dsF: taatacgactcactatagggTGGATCCACGGAGGAGGATT	410
	*LbEST1*-dsR: taatacgactcactatagggATGCCGAACGCTTTCCCTAA	
	*LbEST2*-dsF: taatacgactcactatagggGATCCGTTGAGAGCGGTGAT	383
	*LbEST2*-dsR: taatacgactcactatagggCAAGCCCATGGGTTCAAAGC	
	*LbEST3*-dsF: taatacgactcactatagggGTGCCACTAGCACAAACGTG	321
	*LbEST3*-dsR: taatacgactcactatagggGTATTCCTTCGGGCAGGTCG	
	*LbEST4*-dsF: taatacgactcactatagggTATCCAGCAGGGGAGTTCGT	417
	*LbEST3*-dsR: taatacgactcactatagggCGATTCCGACACCCTGGATT	
	*GFP*-dsF: taatacgactcactatagggCAGTTCTTGTTGTTGAATTAGATG	439
	*GFP*-dsR: taatacgactcactatagggTTTGGTTTGTCTCCCATGATG	

*The reference genes were β*-actin* and α*-tubulin*. The lowercase letters represent the T7 promoter sequences for efficient *in vitro* transcription in dsRNA synthesis.*

cDNA sequences were verified with BLAST and translated using ORF finder.^1^ The catalytic triads and conserved motifs presented in deduced amino acid sequences were identified using InterPro.^2^ The deduced amino acid sequences alignments were carried out using DNAMAN software (DNAMAN 5.2.2; Lynnon BioSoft, Quebec, Canada). Signal peptides were predicted by SignalP 4.1.^3^ The molecular weights and isoelectric points of the amino acid sequences were computed by ExPASy Proteomics Server.^4^ Potential N-glycosylation sites of EST proteins were identified by NetNGlyc1.0 Server.^5^ Phylogenetic tree was constructed with MEGA 7.0 (Kumar et al., 2016) based on the amino acid sequences using the neighbor-joining algorithm. The bootstrap values were calculated on 1,000 replications.

### RNA Interference and Bioassay

**FIGURE 1:**
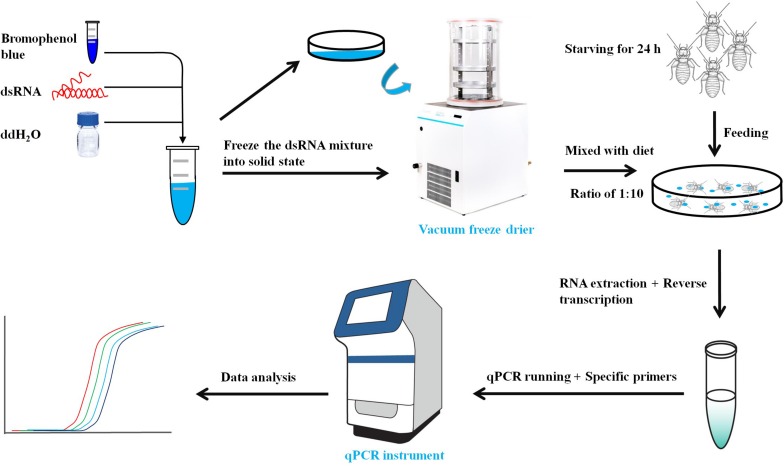
Workflow of Gene knock-down by RNA interference in psocids.

The primers with T7 RNA polymerase promoter were designed to amplify specific genes (Table 1). The dsRNA of *LbEST1*, *LbEST2*, *LbEST3*, and *LbEST4* were synthesized using a Transcript Aid T7 High Yield Transcription Kit (Thermo Fischer Scientific). The protocol of RNAi method in psocids via feeding is as follows (Figure 1): (1) A final of 200 μL mixed liquid that contained 30 μL dsRNA (3–5 mg/mL), 2 μL bromophenol blue and 168 μL RNase-free H_2_O is pipetted into a Petri dish, and then frozen into a solid-state at -80°C refrigerator, (2) The Petri dish with solid mixture was then vacuum freeze dried (Coolsafe 55-4, Labogene, Denmark) for 5 h; (3) The dsRNA dry powder and artificial diet was mixed at a ratio of 1:10, (4) Then 30 adults were fed on this dsRNA containing diet in a plastic vial and then raised under previously described conditions for 1–2 days, and (5) Controls involved feeding with dsGFP, and the silencing efficiencies of the four esterase genes were obtained by qRT-PCR after feeding dsRNA at 24 or 48 h.

In parallel, psocids that survived after RNAi treatment were collected for insecticide bioassays. The malathion bioassay was carried out after 48 h post-feeding of dsLbESTs or dsGFP according to the procedure above. Malathion at LC_1__0_ (5 μg/mL) was applied to test insect sensitivity. Dead insects were recorded at 24 h after malathion treatment. Psocids were determined as dead when they show no response to stimulation by soft brush.

### Quantitative PCR (qPCR)

qPCR was performed using StepOnePlus^TM^ PCR system (Life Technologies, Woodlands, Singapore). The mRNA levels were investigated for the development stages, and insecticide and RNAi treated samples. *Beta-actin* and α*-tubulin* were used as the housekeeping genes to normalize the results of target gene expression (Jiang et al., 2010). The qPCR primers were designed using Primer 3.0^6^ (Table 1). The qPCR reaction (20 μL) contained 10 μL iQ^TM^ SYBR^®^ Green Supermix (BIO-RAD, Hercules, CA, United States), 1 μL of cDNA template, 1 μL of each primer (0.2 mM) and 7 μL of RNase free water. The program was: 2 min at 95°C, followed by 40 cycles at of 95°C for 15 s and at 60°C for 30 s. A final melt-curve step was included (ramping from 60 to 95°C in 0.6°C steps every 5 s) at the end. Relative expression levels were calculated by the 2^–ΔΔ*CT*^ method (Pfaffl, 2001). Each qPCR experiment consisted of three independent repeats.

### Statistical Analysis

Bioassay data were analyzed by SPSS 16.0 for Windows (SPSS Inc., Chicago, United States) using a standard probability value method. The differences in expression levels among different developmental stages were analyzed by one-way ANOVA, followed by Tukey’s test in SPSS 16.0. The significance level was *P* < 0.05. The insecticide-induced expression profiles, changes in enzyme activities and RNAi efficiencies were assessed by Student’s *t*-test (^∗^*P* < 0.05; ^∗∗^*P* < 0.01; ^∗∗∗^*P* < 0.001). All values were represented as mean ± standard error (SE).

## Results

### Synergist Bioassay and Esterase Activity of *L. bostrychophila*

The synergism of TPP, DEM, and DEF toward two insecticides against *L. bostrychophila* was measured (Table 2). Addition of TPP and DEM marginally increased the toxicity of malathion against *L. bostrychophila* with a synergism ratio of 1.66- and 1.56-fold, respectively, whereas DEF showed higher levels of synergism, 5.90-fold. For deltamethrin, only addition with TPP enhanced its lethal effect on *L. bostrychophila*; LC_50_ values were reduced from 4.51 mg/m^2^ to 2.57 mg/m^2^ (synergic ratio = 1.75). However, DEM and DEF showed no synergistic effects on deltamethrin toxicity to this booklouse.

**TABLE 2:** Synergism effect of TPP, DEM and DEF on the toxicity of two insecticides against *Liposcelis bostrychophila*.

**Treatments**	**Slope ± SE**	**LC50**	**(mg/m^2^) (95% CL)**	**χ^2^**	**SR**
Malathion	11.10 ± 1.72	0.53	(0.51–0.55)	3.547	-
Malathion + TPP	6.47 ± 0.69	0.32	(0.30–0.33)	5.479	1.66
Malathion + DEM	2.85 ± 0.31	0.34	(0.31–0.39)	5.758	1.56
Malathion + DEF	2.40 ± 0.28	0.09	(0.08–0.11)	6.370	5.90
Deltamethrin	3.61 ± 0.49	4.51	(4.10–4.88)	2.954	-
Deltamethrin + TPP	2.18 ± 0.27	2.57	(2.23–2.93)	1.375	1.75
Deltamethrin + DEM	3.97 ± 0.52	4.30	(3.89–4.65)	0.658	1.05
Deltamethrin + DEF	2.32 ± 0.33	4.94	(4.25–6.13)	1.878	0.91

*The synergism ratio (SR) indicated that LC_50_ value without synergist was divided by LC_50_ value with synergist. Synergists such as triphenyl phosphate (TPP), diethyl maleate (DEM), and S, S, S-tributyl phosphorotrithioate (DEF) can specifically inhibit carboxylesterases, GST and esterase, respectively.*

Effects of two insecticides on total esterase specific activities *in vivo* are shown in Supplementary Figure S1. After exposure to malathion, the total esterase activities were significantly inhibited at 6 h (α-NA: 0.50-fold, *P* < 0.001; β-NA: 0.58-fold, *P* < 0.01) and then increased at the 12 h (β-NA: 1.15-fold, *P* < 0.01), and finally significantly declined again at 24 h (α-NA: 0.83-fold, *P* < 0.05; β-NA: 0.85-fold, *P* < 0.01) when compared to control. Exposure to deltamethrin, the general esterase activities were suppressed overall the time for both substrates, and they are significantly lower than that in the control at 24 h (α-NA: 0.80-fold, *P* < 0.05; β-NA: 0.81-fold, *P* < 0.05). Exposure to deltamethrin did not substantially affect esterase activities.

### Sequence Analysis and Phylogenetic Tree Construction

**FIGURE 2:**
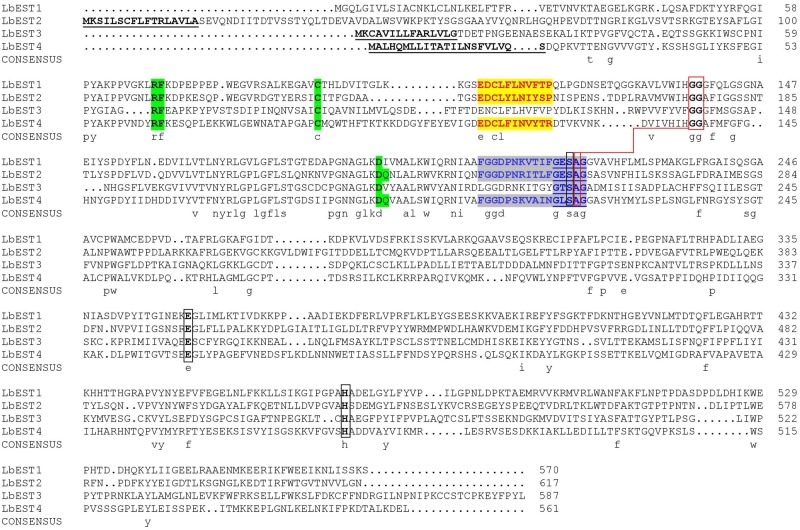
Alignment of the deduced amino acid sequence of four *Liposcelis bostrychophila* esterase genes. The signal peptide is underlined with a solid line in bold letters; the conserved motifs are in green shadow; the catalytic triads are marked with black box; the residues for the oxyanion hole are linked with red box; the esterase conserved motif, GxSxG, is marked with blue letters and underlined with a blue solid line; The type-B carboxylesterase signature 2 motif and serine motif are highlighted in yellow (red letters) and gray (blue letters), respectively.

Based on our previous studies and transcriptome database of *L. bostrychophila*, four putative esterase genes (*LbEST1*-*LbEST4*) that contained complete ORF were identified. The deduced amino acid sequences varied from 561 to 617 aa and the molecular weight ranged from 62.65 to 69.24 kDa (Supplementary Table S1). Multiple alignments of amino acid sequences of *LbEST1*, *LbEST2*, *LbEST3*, and *LbEST4* were conducted (Figure 2). The similarity of amino acid sequences among these four genes is 39.7%. All the four esterase genes have a conserved pentapeptide (Gly-X-Ser-X-Gly), as well as the catalytic triad (Ser-Glu-His) (Figure 2 and Supplementary Figures S2–S5). For *LbEST1* and *LbEST2*, the full-length cDNA consisted of 32 bp and 76 bp 5′ untranslated region (5′UTR), and 304 bp and 595 bp 3′ untranslated region (3′UTR), respectively. Interestingly, a di-nucleotide motif repeat microsatellite (TC)_8_(TT)_2_(TC)_5_ is located on the 3′UTR of *LbEST2* (Supplementary Figure S3). Besides *LbEST1*, the other three genes contained signal peptides and N-glycosylation sites (Supplementary Figures S3–S5).

**FIGURE 3:**
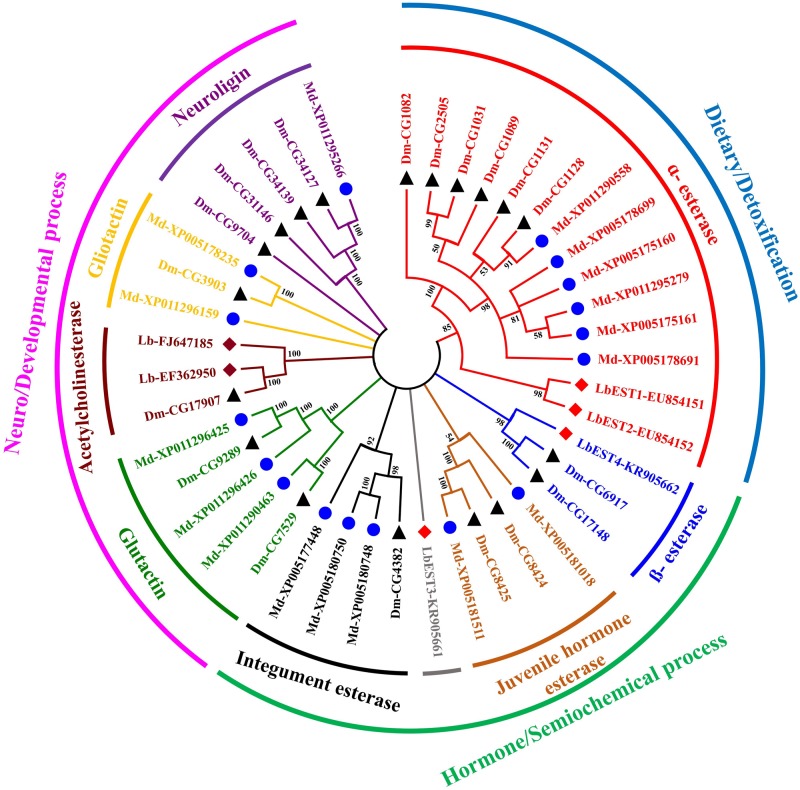
An un-rooted Neighbor-joining tree constructed based on the esterase amino acid sequences from *Liposcelis bostrychophila* and other insects. Esterase genes from *L. bostrychophila* (Lb), *Musca domestica* (Md), and *Drosophila melanogaster* (Dm) are marked by filled rhombus (red color indicates for esterases; brown color indicates for acetylcholinesterases), blue filled circle and black filled triangle, respectively. Different clades are shown in different colors. Nodes with >50% bootstrap values are shown. All insect esterase sequences were retrieved from NCBI.

The constructed phylogeny tree showed that the four *LbESTs* are classified into 4 clades within the two phylogenetic classes, i.e., dietary/detoxification and hormone/semiochemical processing (Figure 3). Phylogenetic analysis with other 38 well-documented esterase genes clearly classified *LbEST1* and *LbEST2* into clade α-esterase, and *LbEST4* is located in the clade of β-esterase. However, *LbEST3* forms a single uncharacterized clade within the class of hormone/semiochemical processing.

### Expressions of *LbESTs* at Different Development Stages and Post-insecticide Exposures

**FIGURE 4:**
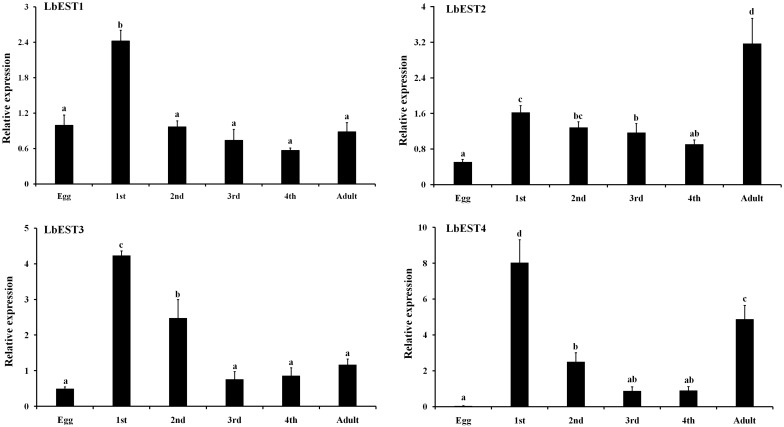
Relative expression levels of *LbESTs* determined by qPCR in different developmental stages. Stage-dependent expression patterns including egg, first, second, third, and fourth-instar nymph, and adults. Bar graph presents values as mean ± SE. Different letters above the error bar for each gene show significant difference (*P* < 0.05).

Developmental expression patterns of four esterase genes were determined in eggs, nymphal instars (1^st^, 2^nd^, 3^rd^, and 4^th^) and adults by qPCR (Figure 4). The results showed that these esterase genes were expressed at all developmental stages, but with stage-specific patterns. Overall, the transcripts of these genes were more abundant in nymphal and adult stages than in the egg. Specifically, the relative expression level of *LbESTs* was significantly higher (over 2.4-fold) in first-instar nymphs compared with those in eggs. The expression of *LbEST2* (6.3-fold) and *LbEST4* (132.9-fold) in adults was significantly higher than those in eggs.

**FIGURE 5:**
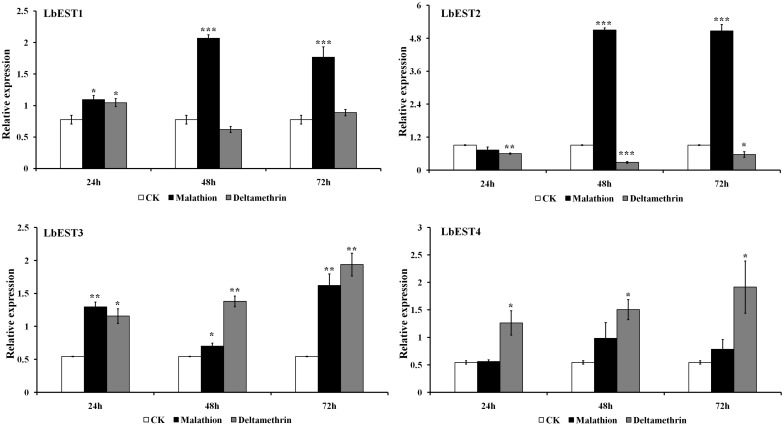
Time course effects of two different insecticides exposure (LC_50_) on the expression profiles of four esterase genes in *Liposcelis bostrychophila*. Bar graph presents values as mean ± SE. Asterisks (*) above the error bars indicate statistical differences determined by the independent samples *t*-test (*P < 0.05; **P < 0.01; ***P < 0.001).

The transcriptional changes of *LbESTs* in adult psocids after exposure to two insecticides were also determined using qPCR (Figure 5). Following exposure to malathion, except for *LbEST4*, the *LbEST1*, *LbEST2*, and *LbEST3* were up-regulated 2. 66-, 5. 60-, and 1.29-fold at 48 h after exposure compared to control, respectively. *LbEST1*, *LbEST2*, and *LbEST3* were also up-regulated 2. 28-, 5. 57-, and 2.98-fold at 72 h after exposure, respectively. For the deltamethrin treatment, the expression levels of *LbEST3* and *LbEST4* were up-regulated, reaching a peak of induction (3.56- and 3.52-fold increase, respectively) at 72 h. However, *LbEST1* and *LbEST2* were down-regulated after different exposure times, and the expression of *LbEST2* was significantly lower (0. 66-, 0. 31-, and 0.62-fold at 12, 24, 72 h after exposure, respectively) than control.

### RNAi and Malathion Bioassay

**FIGURE 6:**
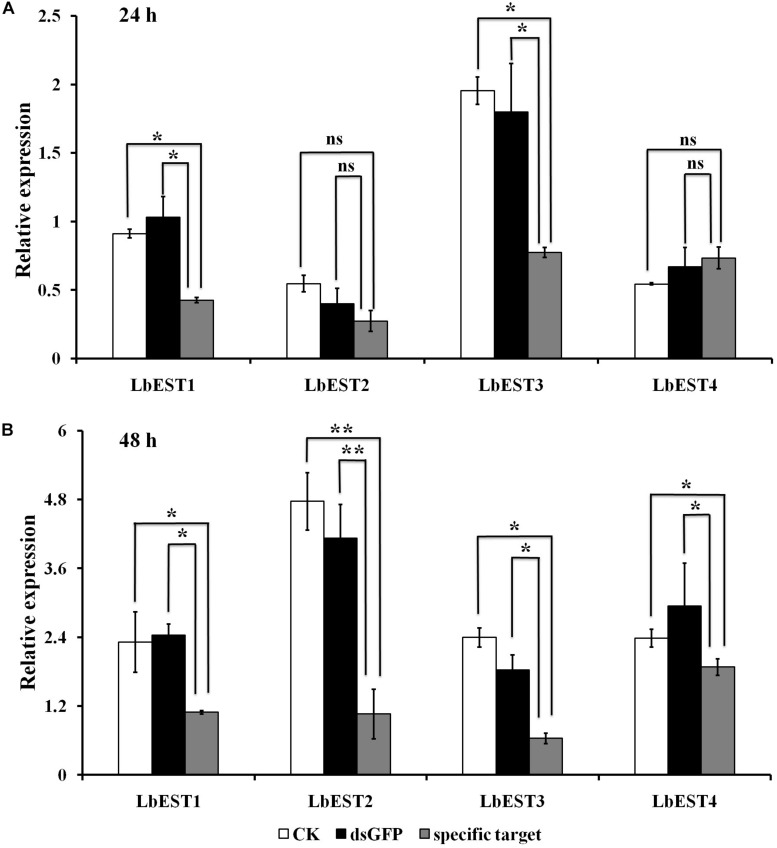
The relative expression of four *LbEST* genes was determined in *Liposcelis bostrychophila* by feeding their corresponding dsRNA. **(A)** The relative expression of four esterase genes in *L. bostrychophila* after feeding their dsRNA separately for 24 h; **(B)** The relative expression of four esterase genes in *L. bostrychophila* after feeding their dsRNA separately for 48 h; GFP-dsRNA was used as the control; Asterisks (*) above the error bars indicate statistical differences determined by the independent samples *t*-test (*P < 0.05; **P < 0.01).

The gene silencing protocol through RNAi in psocids was established for the first time using a non-invasive feeding method (Figure 1). The expression of the four esterase genes decreased remarkably after dsRNA feeding at 48 h, proving that esterase genes could be silenced efficiently by current RNAi delivery system (Figures 6A,B). At 24 h post feeding, the silencing efficiency of *LbEST1* and *LbEST3* was approximately 58.4% and 56.5%, respectively. However, expression of both *LbEST2* and *LbEST4* was not significantly down-regulated, and *LbEST4* was even up-regulated slightly. To further increase the silencing efficiency of these genes, dsRNA feeding time was increased to 48 h. After 48 h of continuous feeding, the silence efficiency of *LbEST3* was elevated to 64.5% (Figure 6B), and the expression levels of *LbEST2* and *LbEST4* (the silencing efficiency was approximately 74.4% and 36.3%, respectively) were significantly decreased from those in control.

After each of *LbESTs* was knocked down, the sensitivity of *L. bostrychophila* to malathion exposure was assessed. The mortality of *L. bostrychophila* with dsLbEST4 feeding slightly decreased compared with that of feeding of dsGFP. RNAi suppression of *LbEST1*, *LbEST2* and *LbEST3* significantly increased the toxicity of malathion (Table 3). The mortality caused by feeding dsLbEST1 and dsLbEST2 was significantly higher than that of feeding dsGFP (13.1%); with 2.54- and 2.69-fold greater, respectively. The mortality following the feeding of dsLbEST3 also increased significantly and was 1.83-fold higher compared with control (Table 3).

**TABLE 3:** The mortality of *Liposcelis bostrychophila* to malathion after *LbESTs* were silenced.

**Treatment**	**Mortality (%) (± SE)**	**n**	**Ratio**
dsGFP	13.10 (± 1.39)^a^	123	
dsLbEST1	33.33 (± 3.03)^c^	118	2.54
dsLbEST2	35.30 (± 0.86)^c^	116	2.69
dsLbEST3	23.97 (± 2.38)^b^	121	1.83
dsLbEST4	11.46 (± 1.21)^a^	126	0.88

*^*a,b,c*^indicate mortality significantly differenced by one-way ANOVA, followed by Tukey’s test at a significance level of 0.05.*

## Discussion

Many studies have revealed that multiple overexpressed EST genes produce more protein to detoxify insecticides (Jackson et al., 2013; Yang, 2016; Xie et al., 2017; Zhang et al., 2018; Wei P. et al., 2019). In this study, we used three synergists (TPP, DEM, and DEF) to investigate the role of one of the major groups of detoxifying enzymes, i.e., esterases, in developing malathion and deltamethrin tolerance in psocids. Overall, pretreatment with the TPP, DEM, and DEF increased malathion toxicity in *L. bostrychophila* (Table 2). These results suggest that ESTs contribute tolerance to malathion in this booklouse (SR = 5.90). Potentially other metabolic enzymes e.g., CarE (SR = 1.66) and GSTs may play a minor role. In deltamethrin exposure, synergist analysis showed that only TPP marginally increased the lethal effect of insecticide on *L. bostrychophila* (SR = 1.75). The current study indicates that ESTs play a minor role in detoxifying deltamethrin in psocids, and other metabolic enzymes, e.g., cytochrome P450 may have a great contribution to tolerance or resistance to this kind of insecticides. A previous study showed that PBO synergized pyrethrins has great potential both as a grain protectant and as a disinfectant against Liposcelidid psocids (Nayak, 2010). Meanwhile, DEF can be potentially used to increase the lethal effect of malathion.

In this study, we characterized four *L. bostrychophila* EST genes designated as *LbEST1*-*LbEST4*, which encode ∼580 amino acids. Among these four genes, *LbEST2*-*LbEST3* might be secreted enzymes, and belong to the N-glycosylated carbohydrate group. The multiple amino acid sequence alignment revealed that *LbESTs* contain typical conserved motifs (Ser-Glu-His and GE (T/L) SAG) for esterases family of proteins, as well as Gly and Ala for the oxyanion hole, which suggests that these *LbESTs* are biologically active (Figure 2). Phylogenetic analysis grouped *LbEST1* and *LbEST2* into α-esterase in the class of dietary/detoxification indicating that they might be involved in metabolic detoxification of pesticides. *LbEST3* and *LbEST4* are located in the clades of β-esterase and juvenile hormone esterase, respectively, suggesting that they might participate in hormone/semiochemical regulation. Furthermore, stage-dependent expression patterns of *LbESTs* show that they were abundantly expressed in 1^st^ nymphal instars and adults with increasing feeding stages when the booklouse likely contact with more xenobiotics. The above results suggest that *LbESTs* may play varying roles in different stages. Many studies documented that ESTs may regulate the growth and development of insects. The specific expression patterns of EST genes in different developmental stages or sexes suggesting their possible functional roles (Campbell et al., 2003; Yang, 2016; Wei L. et al., 2019). Some secreted ESTs (JHE and β-esterase) regulate development or reproduction via regulating the hormone and pheromones signal in insects (Dai et al., 2019).

Intriguingly, it is worth mentioning that a di-nucleotide SSR is detected on the 3′UTR of *LbEST2* (Supplementary Figure S3). Generally, among all the types of SSRs (from di- to hexa-nucleotide repeats), di-nucleotide repeats are the most abundant type of microsatellites in eukaryotic non-coding sequences, but tri-nucleotide repeats account for the majority of microsatellites in coding regions (Li et al., 2002). In some previous studies, di-nucleotide SSRs were also found in the 5′UTR or 3′UTR regions of genes in channel catfish and rat (HSP 70 genes) (Lisowska et al., 1997; Liu et al., 1999). In this study, (TC)_8_(TT)_2_(TC)_5_ is an imperfect microsatellites, which is evolved from (TC)_15_ by two base substitutions (C to T). Based on statistics of EST-SSR from *Liposcelis entomophila* transcriptome, (TC)_n_ accounted for 33.3% of the di-nucleotide repeats (Wei et al., 2013). We infer that (TC)_n_ is also abundant in the gene regions of *L. bostrychophila*. Large amounts of data indicate that mutation of SSR in gene regions can regulate gene function. SSR variations or expansions in 5′-UTRs or 3′-UTRs could regulate gene activity by affecting transcription and translation, even lead to gene silencing or a loss of gene function (Vieira et al., 2016). All the above effects caused by SSR variations in genes can lead to phenotypic changes in organisms (Li et al., 2004). For example, (CT)_n_-SSRs located in the 5′-UTR of tryptophan decarboxylase will influence the promoter activity and thus significantly enhances the level of gene expression in plants (Kumar and Bhatia, 2016). In *Myzus persicae*, the expansion of a di-nucleotide microsatellite in the promoter region of a P450 gene lead to its overexpression, and this process enhanced the resistance to neonicotinoid insecticides in this aphid (Bass et al., 2013).

Induction of ESTs is supposed to be responsible for improving insecticides detoxification, and it may reflect a good compromise between energy-saving and adjustment to a rapidly changing environment (Feng et al., 2018). Our study showed that all *LbESTs* except *LbEST4* can be significantly induced to variable levels after exposure to malathion. Meanwhile, our previous data also showed that the expression of *LbEST2* was more abundant in DDVP and PH_3_-resistant strains in *L. bostrychophila* when compared with susceptible strains (Tang, 2009). Overexpression of esterase genes at transcriptional level have been observed in many insects, which are resistant to organophosphate insecticides (Oakeshott et al., 2005). Many studies confirmed that up-regulation of the esterase genes transcripts and non-synonymous mutations was associated with malathion resistance in insects, suggested that esterase evolution via qualitative and quantitative mechanisms may cause broad insecticide resistance in insects (Zhang et al., 2018). In contrast, *LbEST2* was significantly down-regulated at three time points when exposure to deltamethrin. This result is similar to a recent study, that down-regulation of esterase contributes to cyflumetofen resistance in *Tetranychus cinnabarinus* (Wei P. et al., 2019). Combined with synergistic, phylogenetic and transcripts expression analysis, we inferred that except *LbEST4*, the other three *LbESTs* are recognized as major esterases involved in malathion metabolism in *L. bostrychophila*. Because of this, we only used malathion to test sensitivity changes in this booklouse after the knockdown of the four LbESTs for further verification of the esterase function of insecticide detoxification.

Up-regulated EST genes signaled their importance in the formation of insecticide tolerance or resistance, but how gene over expression connected with phenotype of insecticide susceptibility remains elusive. RNAi is a powerful tool to bridge the gap between gene function and resistance phenotype. Recently, RNAi has been widely applied to study the function of genes in insects, particularly for insects where transgenic technology is insufficient (Kim et al., 2015). However, due to the difficulty of dsRNA delivery, RNAi has not been applied for gene functional studies in psocids. It is impossible to conduct microinjections in booklice without affecting their survival rates, as *Liposcelis* species are very small and soft body insects. Further, artificial diets of psocids are powdery and thus it cannot be mixed with the liquid dsRNA. Also, booklice die easily when they are immersed in or contact water, so soaking or spray methods cannot be applied like in other insects or mites (Marr et al., 2015; Yan et al., 2019). Thus, we established an oral delivery of dsRNA through artificial diets for achieving RNAi in booklice, and this method is suitable and easy to perform for gene functional studies in psocids.

In this study, knockdown *LbEST1*, *LbEST2* or *LbEST3*, the mortality of *L. bostrychophila* was significantly increased after malathion treatment, indicating that these esterase genes are involved in malathion susceptibility (Table 3). Therefore, increased expression of some specific *LbESTs* in *L. bostrychophila* might cause the development of resistance or elevated tolerance to malathion, which may lead to difficulties in control in the field. Our results also indicated that *LbESTs* could be potential RNAi targets in psocids management. However, silencing *LbEST4* shows lower mortality when the psocids were exposed to deltamethrin. Our result is consistent with a previous study that showed knockdown of esterase genes in *Bactrocera dorsalis* also increased the sensitivity of fruit flies to malathion (Wang et al., 2016, 2017). In *P. xylostella*, decreased expression level of EST gene by RNAi could also significantly increase the sensitivity of larvae to the insecticide chlorpyrifos (Xie et al., 2017). In malathion-resistant *Locusta migratoria*, knockdown of carboxylesterases via RNAi would significantly increased susceptibility to malathion treatment (Zhang et al., 2013).

## Conclusion

In conclusion, this study confirmed that esterase genes via up-regulating its expression can mediate decrease of malathion toxicity in *L. bostrychophila.* However, esterases might play limited role in metabolizing the deltamethrin. Thus, overexpression of some specific esterase genes would be one of tolerance or resistance mechanism in *L. bostrychophila*. This is the first report that investigates the role of metabolic enzymes using RNAi in psocids. The establishment of RNAi method in the present study can be used for future research on the functional genes in psocids.

## Data Availability Statement

The datasets generated for this study are available on request to the corresponding author.

## Author Contributions

D-DW, WH, Z-QM, and J-JW conceived the study and participated in its design. D-DW, WH, Z-QM, Y-QT, LW, and WD performed the experiments and analyzed the data. D-DW and WH wrote the manuscript, which all authors edited.

## Conflict of Interest

The authors declare that the research was conducted in the absence of any commercial or financial relationships that could be construed as a potential conflict of interest.

## Footnotes

1 https://www.ncbi.nlm.nih.gov/orffinder/

2 http://www.ebi.ac.uk/interpro/scan.html

3 http://www.cbs.dtu.dk/services/SignalP/

4 http://www.expasy.org/tools/protparam.html

5 http://www.cbs.dtu.dk/services/NetNGlyc/

6 http://frodo.wi.mit.edu/
