# Supplementary material for: Characterization of Esterase Genes Involving Malathion Detoxification and Establishment of an RNA Interference Method in Liposcelis bostrychophila
Source: Front Physiol. 2020 Mar 27;11:274. doi: 10.3389/fphys.2020.00274 (PMC7118802; doi:10.3389/fphys.2020.00274)
Supplement: Supplementary file 1 [file Table_1.doc]

Supplementary Material


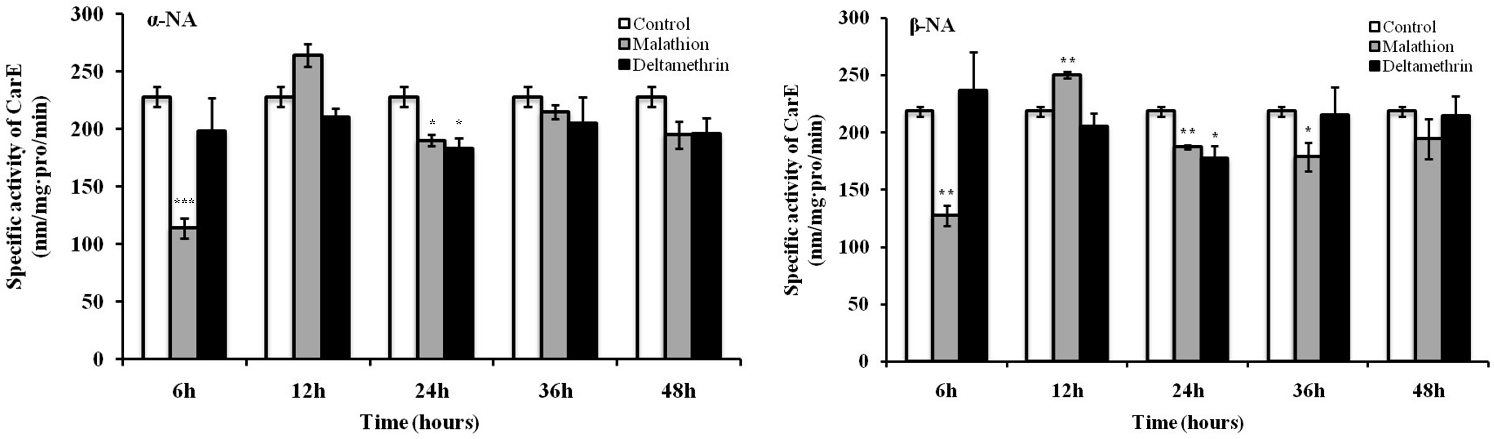


**Supplementary Figure 1.** The total esterase activities of *Liposcelis bostrychophila* exposure to insecticides *in vivo*. The activity was investigated using α-naphthyl acetate (α-NA) and β-naphthyl acetate (β-NA) as model substrate. The asterisks represent statistically significant differences by Student's t-test (**P* < 0.05; ***P* < 0.01; ****P* < 0.001).


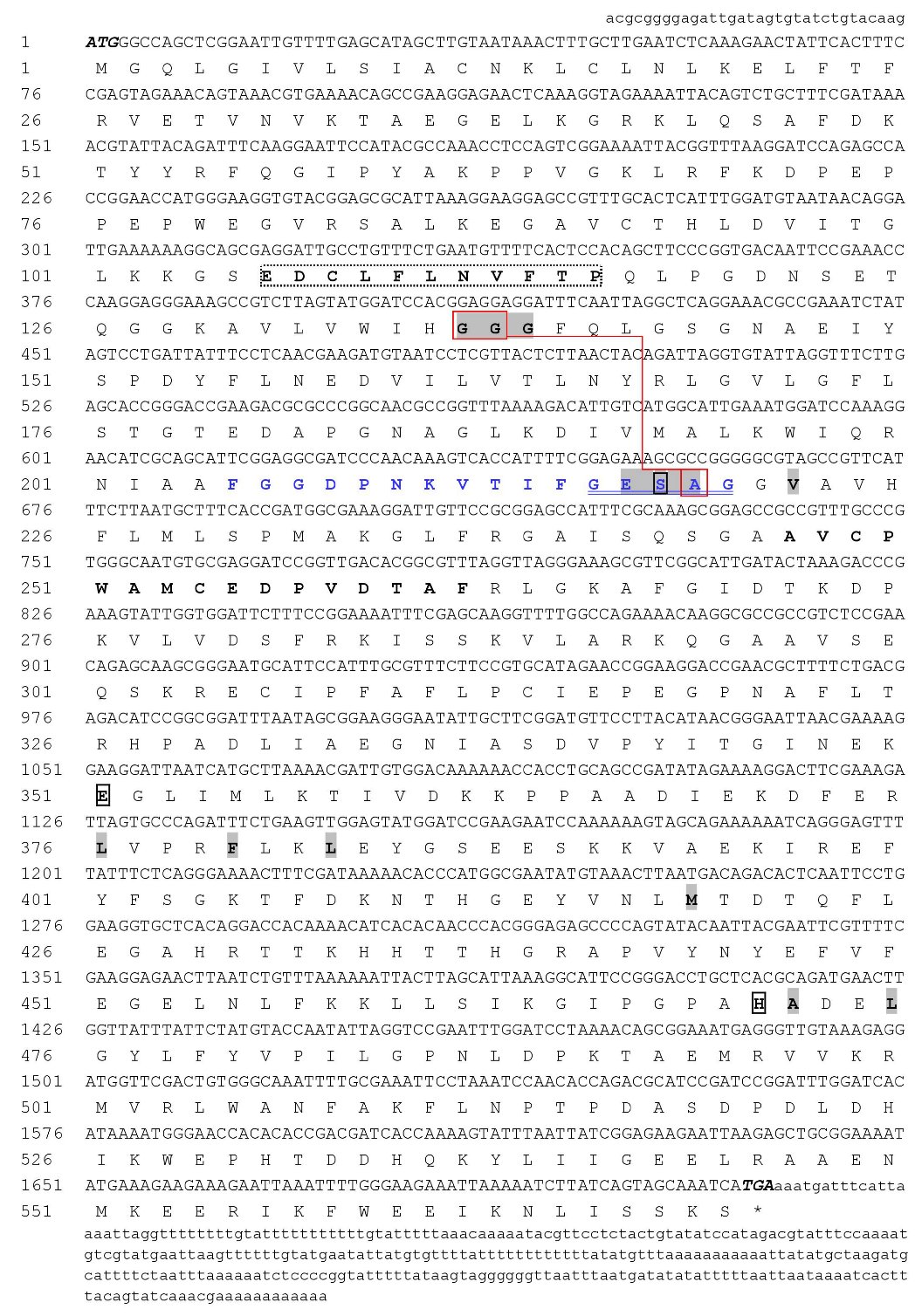


**Supplemenatary Figure 2.** Nucleotide and deduced amino acid sequences of *LbEST1*. The start codon (ATG) and stop codon (TGA) are highlighted in bold and italic; The catalytic triads are marked with black box; The substrate binding pocket are marked letters in grey shadow. In the amino acid sequence, the esterase conserved motif, GxSxG, is underlined with a double line; The carboxylesterase type B, signature sites were marked with dotted box (EDCLFLNVFTP); The carboxylesterase type B, active site was marked with blue letters (FGGDPNKVTIFGESAG); The residues for oxyanion hole are linked with red box.


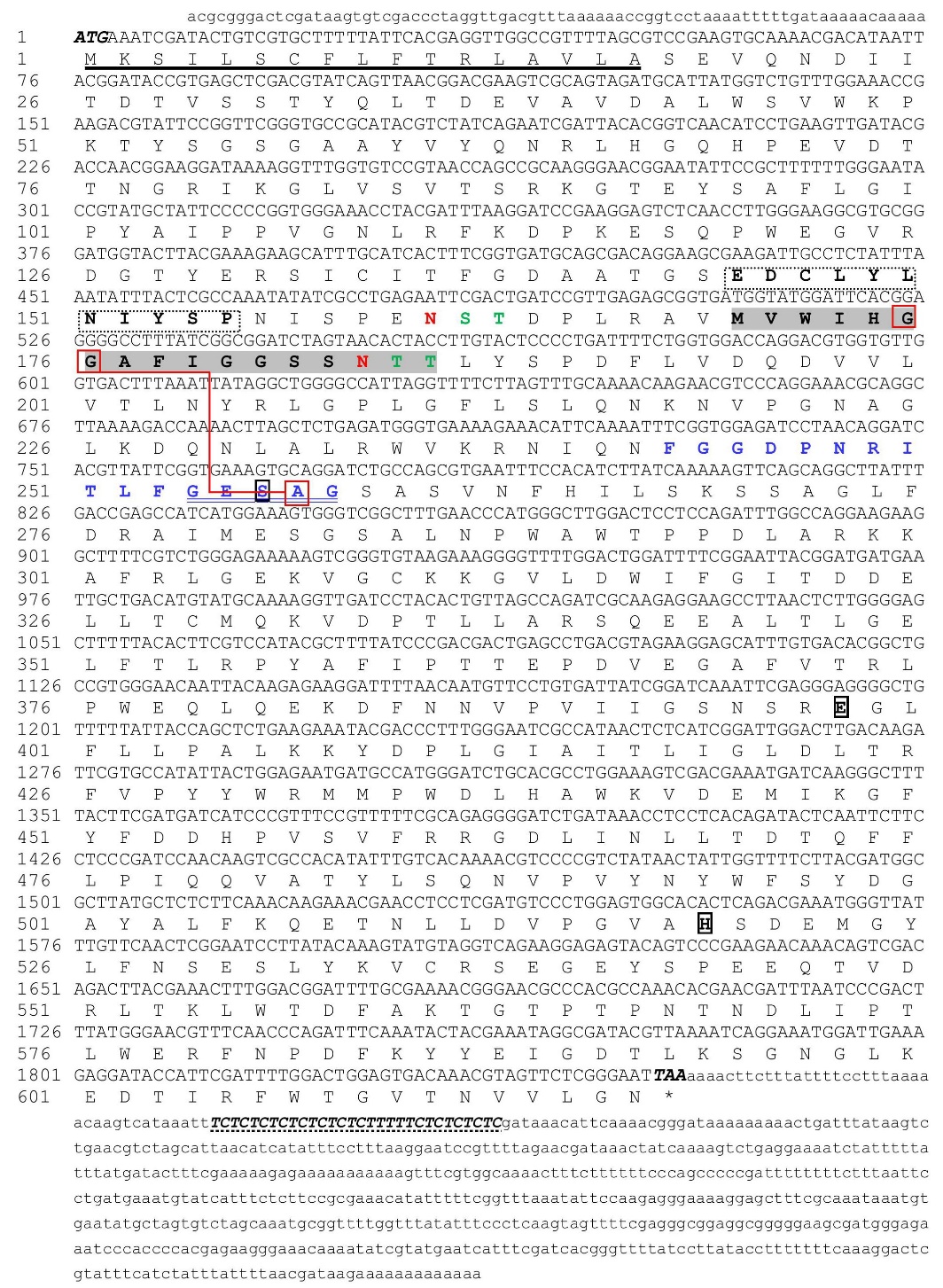


**Supplementary Figure 3.** Nucleotide and deduced amino acid sequences of *LbEST2*. The start codon (ATG) and stop codon (TAA) are highlighted in bold and italic; The signal peptide is underlined with a solid line; The catalytic triads are marked with black box; The substrate binding pocket are marked letters in grey shadow. In the amino acid sequence, the esterase conserved motif, GxSxG, is underlined with a double line; The carboxylesterase type B, signature sites were marked with dotted box (EDCLYLNIYSP); The carboxylesterase type B, active site was marked with blue letters (FGGDPNRITLFGESAG); Lipase, GDXG, putative histidine active site were marked in grey shadow (MVWIHGGAFIGGSSNTT); The residues for oxyanion hole are linked with red box; Asparagines predicted to be N-glycosylated are shown in red and Asn-Xaa-Ser/Thr sequons (including Asn-Pro-Ser/Thr) are shown in green; The microsatellite locus (TC)_8_(TT)_2_(TC)_5_ is underlined in a dotted line at the 3’ untranslation region of *LbEST2*.


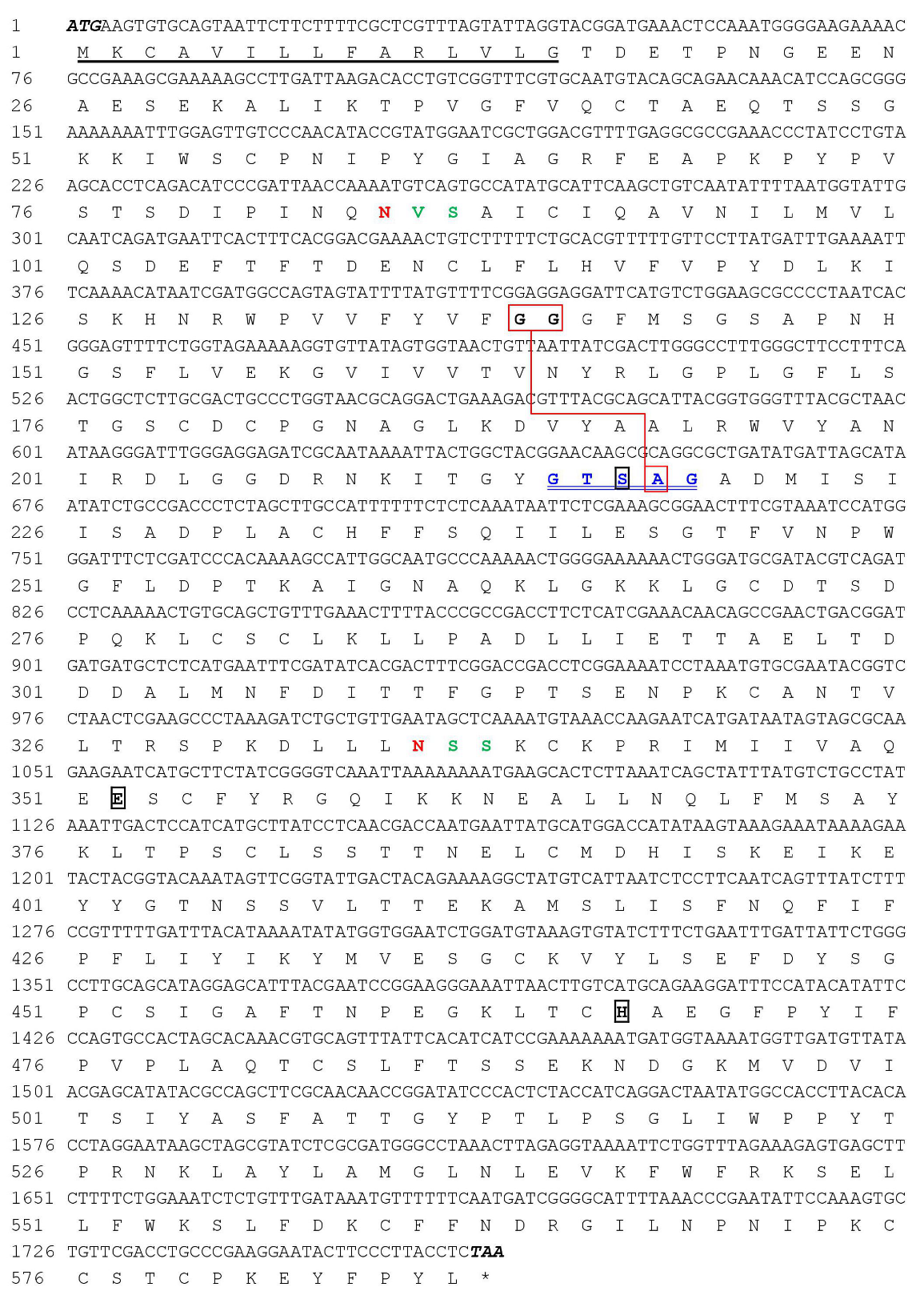


**Supplementary Figure 4.** Nucleotide and deduced amino acid sequences of *LbEST3*. The start codon (ATG) and stop codon (TAA) are highlighted in bold and italic; The signal peptide is underlined with a solid line; The catalytic triads are marked with black box; In the amino acid sequence, the esterase conserved motif, GxSxG, is underlined with a double line; The residues for oxyanion hole are linked with red box; Asparagines predicted to be N-glycosylated are shown in red and Asn-Xaa-Ser/Thr sequons (including Asn-Pro-Ser/Thr) are shown in green.


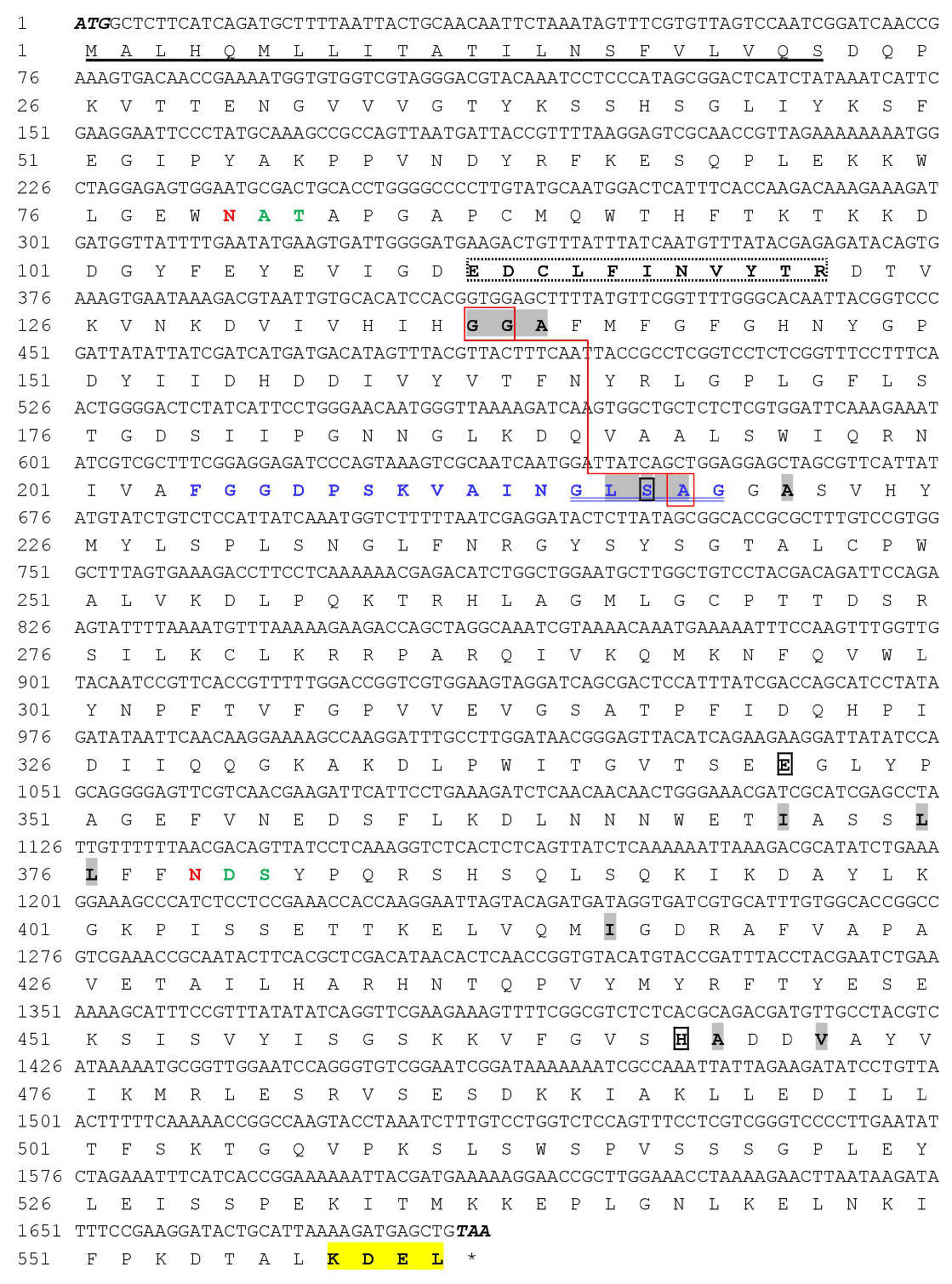


**Supplementary Figure 5.** Nucleotide and deduced amino acid sequences of *LbEST4*. The start codon (ATG) and stop codon (TAA) are highlighted in bold and italic; The signal peptide is underlined with a solid line; The catalytic triads are marked with black box; The substrate binding pocket are marked letters in grey shadow. The esterase conserved motif, GxSxG, is underlined with a double line; The carboxylesterase type B, signature sites were marked with dotted box (EDCLFINVYTR); The carboxylesterase type B, active site was marked with blue letters (FGGDPSKVAINGLSAG); The residues for oxyanion hole are linked with red box; Asparagines predicted to be N-glycosylated are shown in red and Asn-Xaa-Ser/Thr sequons (including Asn-Pro-Ser/Thr) are shown in green; Endoplasmic reticulum targeting sequence (KDEL) is highlighted in yellow shadow.

**Supplementary Table 1. Overview of molecular properties of four esterase genes in *Liposcelis bostrychophila*.**

| Gene name | Genbank No. | ORF (bp) | No. of AA | MW (kDa) | pI |
| --- | --- | --- | --- | --- | --- |
| *LbEST1* | EU854151 | 1713 | 570 | 63.36 | 6.53 |
| *LbEST2* | EU854152 | 1854 | 617 | 69.24 | 5.07 |
| *LbEST3* | KR905661 | 1764 | 587 | 64.89 | 6.16 |
| *LbEST4* | KR905662 | 1686 | 561 | 62.65 | 7.64 |
